# Phlebotomine sand flies in Southwest Germany: an update with records in new locations

**DOI:** 10.1186/s13071-020-04058-6

**Published:** 2020-04-21

**Authors:** Sandra Oerther, Hanna Jöst, Anna Heitmann, Renke Lühken, Andreas Krüger, Irmgard Steinhausen, Christine Brinker, Susanne Lorentz, Michael Marx, Jonas Schmidt-Chanasit, Torsten Naucke, Norbert Becker

**Affiliations:** 1 Institute of Global Health, Heidelberg University, Heidelberg, Germany; 2 German Mosquito Control Association (KABS), Speyer, Germany; 3 Institute for Dipterology (IfD), Speyer, Germany; 4 Bernhard-Nocht-Institute for Tropical Medicine, Hamburg, Germany; 5 Friedrich-Loeffler-Institut, Federal Research Institute for Animal Health, Greifswald-Insel Riems, Germany; 6 Faculty of Mathematics, Informatics and Natural Sciences, University Hamburg, Hamburg, Germany; 7 Bundeswehr Hospital Hamburg-Branch Tropical Microbiology & Entomology, Hamburg, Germany; 8 Parasitus Ex e.V., Niederkassel, Germany; 9 Laboklin GmbH & Co. KG, Bad Kissingen, Germany

**Keywords:** Sand flies, Germany, Field study, Spatial distribution, *Phlebotomus mascittii*

## Abstract

**Background:**

Vector-borne diseases (VBD) are of growing global importance. Sand flies are potential vectors for phleboviruses (family *Phenuiviridae*) including Toscana virus (TOSV), Sicilian virus, Sandfly fever, Naples virus, and *Leishmania* parasites in Europe. To date, only two phlebotomine species have been recorded for Germany: *Phlebotomus perniciosus* and *Phlebotomus mascittii*. This study updates the distribution and abundance of the two occurring species.

**Methods:**

An entomological field study was carried out during 2015–2018 to assess the abundance of sand flies in Southwest Germany within the federal states Baden-Wuerttemberg (BW) and Rhineland-Palatinate (RLP). A total of 176 collection sites were examined using CDC light traps.

**Results:**

A total of 149 individuals of *P. mascittii* were collected. During 2015–2018, *P. mascittii* was found at all sites known positive from previous studies and was detected at 15 additional sites previously unknown for the presence of sand flies. Although the environment has changed considerably in 30 years, no significant difference in sand fly dynamics and distribution was found. *Phlebotomus perniciosus* has only been trapped once since 2001.

**Conclusions:**

This study showed that sand flies occur in different areas in Southern Germany where they had not been recorded previously. Therefore, it can be assumed that they are more widespread than expected. In addition, sand flies could be found over several years at the same trapping sites, indicating population stability. This supports the need for continued surveillance of possible vector populations and urgent clarification of the vector competence of *P. mascittii*.
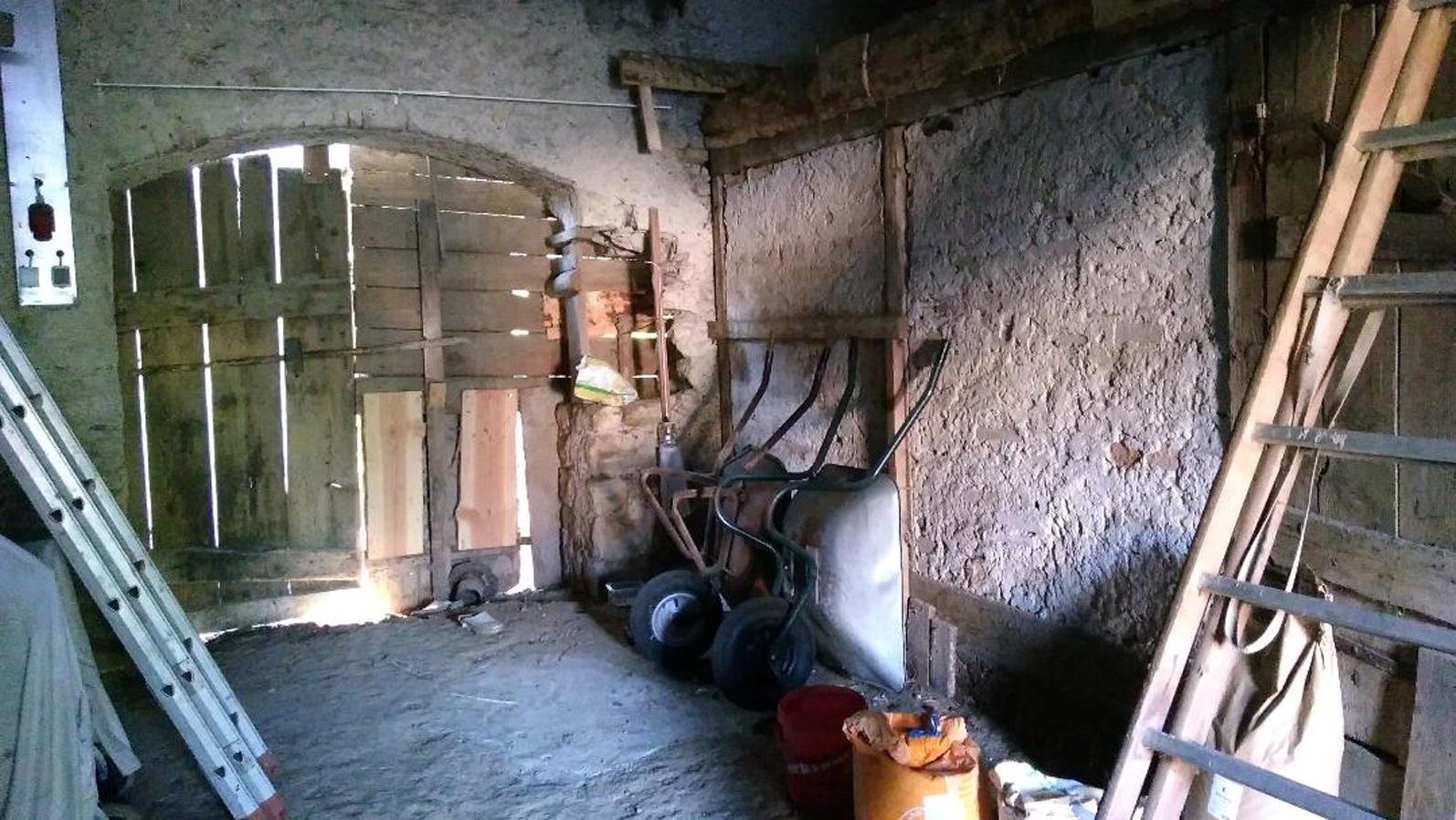

## Background

Sand flies (Diptera: Psychodidae: Phlebotominae) are insects with more than 1000 species worldwide. Species of the genus *Phlebotomus* are distributed in the Old World and species of *Lutzomyia* are distributed in the New World [1]. The vector group occurs throughout the tropics and sub-tropics, as well as in temperate zones. In Europe, sand flies are widespread in the Mediterranean area with about 25 species known to be present [2]. However, with increasing temperatures due to climate change, species such as *Phlebotomus ariasi* Tonnoir, 1921 and *Phlebotomus perniciosus* Newstead, 1911 are predicted to extend their range [3, 4].

In Germany, sand flies were detected for the first time in July 1999 during the first monitoring study in southwest Baden-Wuerttemberg (BW) [5]. Four specimens of *Phlebotomus mascittii* Grassi, 1908 were caught at three locations along the Upper Rhine Valley. In the following years the investigations were extended. By 2007, 237 specimens of *P. mascittii* had been found at 17 locations in different studies [6–8]. Additionally, in 2001, four specimens of the *P. perniciosus* were recorded for the first time in Germany and to our knowledge the species has never been trapped since then [8]. It seems that the collection sites of *P. perniciosus* and *P. mascittii* differ, since the trapping site of *P. perniciosus* was different from the locations where the *P. mascittii* specimen was caught in the latter study [8].

Sand flies in Germany are mostly active during the warm summer months, which is described in southern Switzerland, with climate conditions similar to those in the area investigated in the present study [9]. Female sand flies feed on different vertebrate hosts including humans, livestock, dogs, rodents, reptiles, amphibians and birds [10]. Host availability seems to be an important factor determining feeding behaviour. The main hosts of *P. mascittii* are dogs and humans, while *P. perniciosus* prefers dogs, humans, horses and rodents [11]. In addition, *P. mascittii* is known to also have an autogenous feeding behaviour, sucking on plant and fruit juices [12].

*Phlebotomus perniciosus* is a vector for parasitic protozoa (*Leishmania* spp.) [13] and viruses [14]. In Europe leishmaniasis is the most common sand fly-transmitted disease, caused by two parasites, *Leishmania infantum* and *Leishmania tropica*, causing visceral leishmaniasis and cutaneous leishmaniasis, respectively. In the Mediterranean basin, visceral leishmaniasis is the main form of disease, while cutaneous leishmaniasis arises periodically in Greece and surrounding countries [15]. In 2001, one case of human leishmaniasis was confirmed in Germany in a child that had no history of travel to known endemic areas [16]. One equine case [17] and several canine cases [18] have been described where the case history could indicate autochthonous infection within Germany, suggesting that infected sand flies must be present at least periodically. To date, *P. mascittii* has not yet been confirmed as a vector of leishmaniasis*. Leishmania infantum* was detected in a non-engorged female *P. mascittii* in Austria, close to the border of Slovakia [19]. On the island of Montecristo, Italy, *Leishmania* parasites could be found in rats in association with *P. mascittii* [20]. These findings suggest that *P. mascittii* could be relevant in leishmaniasis epidemiology.

*Leishmania* parasites are regularly imported by dogs and humans from endemic regions to Germany leading to a continual risk of autochthonous transmission [21]. Tourists return to Germany with infected dogs from the Mediterranean area and several animal welfare organizations support importing dogs from the Mediterranean region [6]. According to current estimates, there are already more than 100,000 leishmaniasis-positive dogs present in Germany [22]. However, the risk of human infection in Germany is still expected to be extremely low.

Sand flies are vectors for phleboviruses (family *Phenuiviridae*) including Sandfly fever Naples virus and Sandfly fever Sicilian virus. Sand fly-borne phleboviruses are widely distributed in the Mediterranean region and mainly cause mild disease characterized by fever, myalgia and headache. Several new phleboviruses have been detected in Europe in the last decade [23–26]. However, the medical and veterinary importance of these newly described viruses is yet to be investigated. To date there is no convincing evidence that a species of vertebrates is a natural reservoir of phleboviruses. The natural cycle of phleboviruses is poorly understood [27]. Neuroinvasive infections were reported for Sandfly fever Naples virus in Italy, Spain, Portugal and Cyprus [28, 29].

Case reports of seroepidemiological studies showed that the geographical distribution of Toscana virus is much wider than previously assumed and despite its importance as a human pathogen, it remains neglected and there are consequently many gaps of knowledge [30]. Especially during outdoor activities, the exposure to sand flies can increase the risk of an infection with phleboviruses [31].

In 2010, human Toscana virus infections were detected in 6.6% (*n* = 150) of the cases diagnosed as meningitis or encephalitis of unknown aetiology in southern Germany [32].

In at least one of these cases, the affected individual had never left the area, so autochthonous transmission must be assumed [33].

Temperature plays an important role in the distribution of sand fly species and global warming will lead to an extension of the distributional area and thereby also to an increase in the risk of sand fly-borne infections [34]. Since 2008, no active field work has been carried out in Southwest Germany. Therefore, the here presented field study aimed to collect new data about the current distribution, occurrence and abundance of sand flies in this area.

## Methods

### Study area and survey

**Fig. 1:**
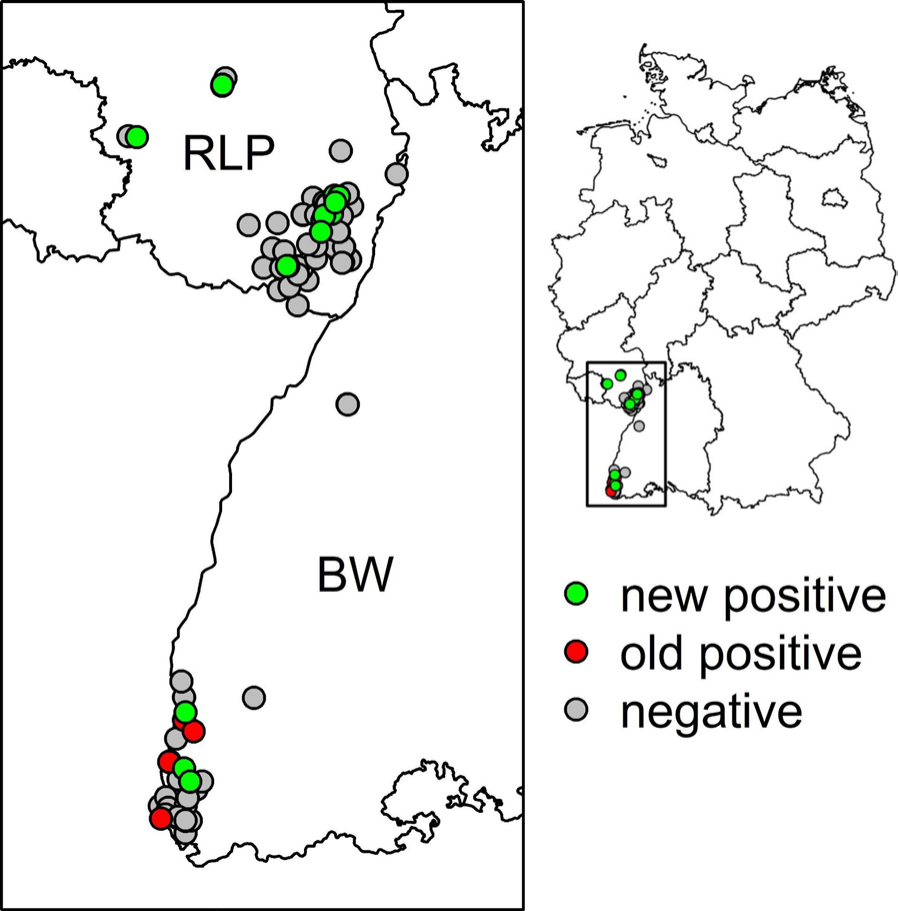
Map of collection sites for sand flies in Southwest Germany (BW, RLP) during 2015–2018. *Key*: old positive sites (red); negative sites (grey); new positive sites (green)

A longitudinal study on the distribution of sand flies along the Upper Rhine Valley was carried out in the federal states Baden-Wuerttemberg (BW) and Rhineland-Palatinate (RLP) between 11th and 21st August 2015 and during June-September 2016–2018 (Fig. 1). The number of trap nights was 108, 74, 415 and 272 in the years 2015–2018, respectively. In 2015, based on previously known sites with established sand fly populations, six collection sites were chosen. Two of these were entirely new sites, selected in close proximity to 4 other known sites. In the following years, the selection of sites was done according to the following two conditions: sites already studied in previous studies, and newly selected sites with potentially favourable environmental and climatic conditions, i.e. having three consecutive nights with a minimum temperature of 15 °C. In order to find new locations, citizens were informed about the study with an information flyer. In addition, local health authorities and municipalities were informed, and advertisements made in local newspapers. Citizens were asked to report potential sand fly trapping sites, the required conditions of which were described in the flyer.

All locations were properly inspected, and habitat types were identified (animal shelters, farms, old quarries, caves) according to standard protocols [35]. In 2017, the area was extended to RLP, the area where *P. perniciosus* was trapped in 2001. In 2018, only positive sites were sampled with increased frequency, with the intent to catch higher numbers of sand flies for pathogen screening. Temperature data from the study area were collected from the German Meteorological Service (DWD) [36]. Mean annual climatic characteristics from 2015 to 2018 of both study areas can be summarized as follows: annual minimum temperatures of − 13.9 °C, annual maximum temperatures of 35 °C, mean summer temperature of 21.5 °C (June to September), summer precipitation of 200–250 mm [36].

### Sand fly sampling

Collection was done with Center for Disease Control and Prevention miniature light traps (CDC miniature light traps 512, Bioquip, CA, USA). Depending on the size of the collection site 1 to 5 CDC traps were deployed with a distance of about 5 m from each other and operated between 18:00 h and 8:00 h. Traps were predominantly placed inside barns, close to the ground or walls with or without organic material at a height of 1–1.5 m. The placement of the traps was selected according to previously described habitat preferences of sand flies [6]: old barns with non-concrete ground, brick or mud walls, often abandoned and sheltered from wind [5]. Global positioning system (GPS) coordinates and environmental descriptions were recorded on site. In 2018, temperature and humidity were recorded in both study regions (BW and RLP) with Hobo Pro v2 data loggers at 12 different locations (Additional file 1: Table S1) from June to the end of September (Onset, MA, USA). In each state, 6 sites were chosen, according to the highest capture rate. Depending on the capture rate, collections were repeated up to 9 times during one season, with the aim of capturing high numbers of sand flies (Additional file 1: Table S1). Captured insects were immediately stored in a polystyrene box with cool packs and manually inspected on a sheet of white paper to eliminate bycatch. Afterwards, sand flies were preserved in 100% ethanol and kept in 1.5 ml reagent tubes. A sand fly trapping factor (number of sand flies/trap-night) was calculated to compare the numbers of sand flies trapped at each collection site. Trap-nights are defined as the number of sampling nights multiplied with the number of traps per site.

### Molecular identification and pathogen screening

Each specimen was processed individually. Deoxyribonucleic acid (DNA) and ribonucleic acid (RNA) was extracted with a KingFisher™ Flex Magnetic Particle Processor using MagMAX™ Pathogen ribonucleic acid/DNA Kit (Thermo Fisher Scientific, Waltham, MA, USA). Morphological identification of sand fly species was confirmed by DNA barcoding analysis of the cytochrome *c* oxidase subunit 1 (*cox*1) as described by Polseela et al. [37]. Polymerase chain reaction (PCR) products were subjected to Sanger sequencing. The resulting sequences were submitted for species identification using the basic alignment search tool (BLAST) in the GenBank DNA sequence databases (https://blast.ncbi.nlm.nih.gov/). For virus screening, extracted RNA samples were tested by pan-phlebovirus [38] and pan-flavivirus PCR [39].

In 2018, the 81 specimens were screened on *Leishmania* parasites with a quantitative polymerase chain reaction (qPCR) using the primers OWLeishkDNAfwd* (3′-GCT TTA GTG GGT TGG AGC CT-5′), OWLeishkDNArev* (3′-TCA ACC CAA GAC CAC CAT CG-5′) and OWLeishkDNAProbe*: (6FAM)CGG GTG TCT TTG ATG ATG CTG GGG TGG GT(BHQ1) [40]. The qPCR was performed with the SensiFAST™ Probe No-ROX Kit (Bioline) with 10 µl Master Mix Bioline (2×), 0.3 µl of each primer (900 nM), 0.5 µl probe (200 nM), 1.2 µl MgCl_2_ (25 mM), 1.7 µl distilled water and 6 µl DNA template. The reaction was conducted with denaturation for 5 min at 95 °C followed by 37 cycles at 95 °C for 10 s, 57.5–66.0 °C for 40 s (touchdown 0.5 °C for 17 cycles) and a final elongation for 30 s at 40 °C. As a positive control, genomic DNA isolated from one sand fly (*P. perniciosus*) with added 200 *Leishmania infantum* strain 3511 parasites was used. The detection minimum for the parasite was determined as 50 *L. infantum* parasites per sand fly.

## Results

During 2015–2018, a total of 149 (92 female and 57 male) sand flies were collected at 37 (21%) out of 176 collection sites (Table 1). The earliest trap night was on the 30th June and the latest trap night on the 21th September. The earliest capture of sand flies during the entire study period was on the 3rd of July 2018 and the latest on the 31th of August 2017 (Additional file 1: Table S1). All sand fly specimens were identified as *P. mascittii* by morphology and barcoding of the *cox*1 gene fragment. None of the trapped sand flies were engorged.

**Table 1:** Prevalence of sand flies from collection sites in Southwest Germany during 2015–2018

Year	Trapping period	No. of collection sites	No. of sampling nights	No. of trap nights	No. of collected sand flies (female/male)	Sand fly trapping factor
2015	11–21 August	6	8	108	16 (9/7)	0.15
2016	22 July to 29 August	36	9	74	0	0
2017	18 July to 31 August	100	31	415	52 (30/22)	0.13
2018	29 June to 21 September	34	31	272	81 (53/28)	0.30
Total		176	79	869	149 (92/57)	0.17

The sand fly trapping factor over the entire sampling period indicates that 0.17 sand flies were caught per trap on average. The highest trapping factor was found for the year 2018 with 0.3 sand flies per trap. In 2015, 16 specimens (7 male and 9 female) of *P. mascittii* were caught at 5 (83.3%) out of 6 sampling sites in BW. Two new collection sites were identified in this year. In summer 2016, no sand flies were recorded at 36 new, previously uninvestigated collection sites. In 2017, 52 *P. mascittii* (22 male and 30 female) were captured. Out of 100 collection sites, 17 (17%) were positive for phlebotomine sand flies. Two new sites in the federal state BW and 10 new sites for the federal state RLP were identified. In addition, 5 previously known collection sites for *P. mascittii* for BW were confirmed. In 2018, 81 *P. mascittii* (28 male and 53 female) were trapped. Out of 34 collection sites, 15 (44%) were confirmed positive. One new collection site was found positive for RLP. Within the duration of the study, 15 new sites infested with sand flies were identified.

Overall, the percentage of females was 60.3%. Only in July 2017, the percentage of males was higher at 54.2%. In other months the percentage of females was between 56.2–67.9%.

From late August, the number of sand flies captured decreased, until early September, where none were captured. It was observed that the number of sand flies decreased after several sampling nights at a collection site, especially at sites with high sand fly trapping numbers (Additional file 1: Table S1).

**Fig. 2:**
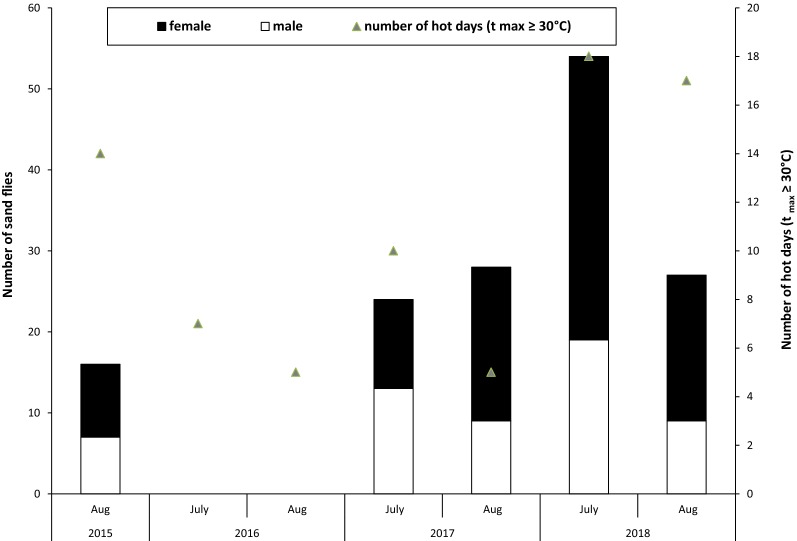
Number of sand flies collected in Southwest Germany during 2015–2018 and number of hot days (t_max_ ≥ 30 °C) (triangles)

The mean temperature of the months July and August in 2015, 2016, 2017 and 2018 was 22.9 °C, 21.4 °C, 21.3 °C and 23.1 °C, respectively. In July 2018, 18 days were hot days with temperatures above 30 °C. The month with the fewest hot days was August 2016 with 7 hot days (Fig. 2). Temperatures and relative humidity recorded from the data logger in 2018 were analyzed. At Bremgarten, a site where sand flies were caught regularly, temperatures on the trapping days at 19:00 h ranged from 27.7 °C to 30.8 °C with a relative humidity between 35.8–48.3%. Sites negative for sand flies did not differ significantly in humidity and temperature.

The most common trapping sites were old, natural barns, especially those with non-concrete soil, in the immediate vicinity of humans and animals such as dogs, cats, rats, horses, chickens and lizards. They were close to human dwellings and generally characterized by old, uneven, and cracked brick walls with moist soil and tamped clay. In addition to this, they were often quiet and abandoned. Six *P. mascittii* were found in a sylvatic site named “Isteiner Klotz”. Seven other sylvatic sites (caves in forestry areas) were investigated in 2017 but they were all negative for the presence of sand flies.

All sand flies tested negative for phleboviruses, flaviviruses and *Leishmania* spp. infection.

## Discussion

In this study, sand flies were trapped at 37 out of the 176 sites in Southwest Germany. This shows that these potential vectors are more widespread than previously known. However, non-detection of sand flies at a trap site should be interpreted with caution and not taken immediately as evidence of a lack of a population there. Sites which had regularly been positive in previous studies were positive for sand flies again, demonstrating stable populations at these sites. The number of trapped sand flies was always very small, usually only 1 or 2 specimens. In most studies, *P. mascittii* is found in low population densities [41–44]. The low density may be due to the specific ecological niche they are adapted to (caves, tunnels and natural barns) [41, 45]. Generally, CDC light traps are used to determine relative changes in abundance over time and space [46], but for species like *P. mascittii* capture methods have to be adapted as they are probably less attracted by light and carbon dioxide [9]. It is even suspected that *P. mascittii* is “light-shy” and that the catches are possibly only random catches [47]. In addition, using pieces of fruit as further attractants for *P. mascittii* has been suggested by Poeppl et al. [48] as a way to increase trapping effectiveness. In order to make precise predictions about the distribution of sand flies in Germany, trapping must be extended to other areas using different methods.

*Phlebotomus mascittii* is widely distributed in central Europe and can be found north of the Alps in countries like France [41], Switzerland [9] and Belgium [49]. In Germany, this species was found in the states of BW and RLP, while in other regions no sand flies have been detected to date. The state of Bavaria in Southern Germany was investigated in previous studies for sand fly presence, but negative for sand flies. Furthermore, since the first detection of *P. mascittii* in Germany, CDC traps for sand fly trapping were placed sporadically along the Rhine plane in the state North Rhine Westphalia (NRW) up to Duesseldorf over the past 20 years but have thus far been negative for sand flies (TN).

With the assumption that sand flies are remnants of the Holocene, they have survived in small refugial areas and probably could be found in other regions of Germany. With global warming and climate change they might expand their range and disperse to areas where suitable habitats are available [3]. Generally, sand flies require a temperature of at least 20 °C during the warmest month and an annual mean temperature of 10 °C [50, 51]. Consequently the 10 °C-annual-isotherm is regarded as the northern boundary for sand fly distribution, which has already shifted north in the last decades [34]. During the study period, monthly mean temperatures were between 21.3 °C and 22.9 °C. Therefore, temperatures could probably not explain the negative trapping results in 2016. However, there were fewer hot days (tmax ≥ 30 °C) and more periods with rain in 2016 compared to the other years. Rainfall reduces the amount of suitable diurnal resting sites for the adult insects and limits the adult flight activity, leading to lowered trapping success [52]. The deviating weather conditions could also have led to smaller population densities. Many obscure factors such as sferics, humidity, air pressure changes, and other weather phenomena likely have an impact on sand fly abundance [53]. Even some trap sites where sand flies were caught regularly in previous years were negative.

Results from France and Switzerland suggest that *P. mascittii* only undergoes one generation per year in temperate regions [9]. This has previously been shown for Germany [54]. Generally, sand flies have a longevity of 7 to 10 weeks, with cooler temperatures leading to decreased longevity [7]. Our data suggest that only one generation is developing, as the number of trapped sand flies decreased at the end of August (Additional file 1: Table S1). The number of females captured in our study was higher on average than the number of males; this could be seen also in other studies [2, 8]. Under laboratory conditions the proportion of males to females is 50:50 [7]. Females are probably more attracted by the CO_2_ as they search for a host or breeding site. In addition, compared to females, males have reduced flight activity and spend more time resting. This phenomenon reduces the likelihood of male sand flies being caught in a trap.

Remarkable was the finding of six individuals within the only known sylvatic site named “Isteiner Klotz” in 2018. For 17 years, no sand flies had been detected there, despite trapping occurring on several occasions. The site lies within a forest and sand flies were caught in a small cave within a rocky outcrop. Several sylvatic sites similar to the “Isteiner Klotz” were investigated in 2017. No further sites were positive for sand flies. Outside Germany, *P. mascittii* is known to be found in such ecological niches, where temperature and humidity remain more constant than in open environments [45].

Pathogens transmitted by phlebotomine sand flies are already present in Germany and transmission of *Leishmania* parasites and phleboviruses has been suspected [17, 33]. To date it has not been proven that *P. mascittii* is a vector for *Leishmania* parasites. *Leishmania infantum* has been detected in *P. mascittii*, but transmission could not be confirmed (TN, unpublished data). There are few studies on the vector competence of sand flies as their colonisation is difficult [55, 56]. Nevertheless, several criteria for the possible vector capacity of *P. mascittii* are fulfilled, such as anthropophilic behaviour and feeding on reservoir hosts. More than 100,000 *Leishmania*-infected dogs are currently living in Germany and the number of dogs imported to Germany from endemic areas is growing, leading to an increase in cases of leishmaniasis [23]. The risk for a human autochthonous *Leishmania* infection [47] or the formation of *Leishmania* cycles in Germany is so far very low. In addition, sand flies tested in our study were all negative for phleboviruses and flaviviruses. However, the low number of sand flies tested does not rule out circulation of these viruses in Southwest Germany [17]. Further studies are required to evaluate if these pathogens circulate in the sand fly populations in Germany. This situation might change in future if sand fly species expand in range due to climate change and build up larger populations [30]. In addition, further studies are necessary to clearly characterize the environmental conditions of the habitats of *P. mascittii.*

## Conclusions

The study results reveal that sand flies occur in different areas where they have not been detected before. During the study, 15 new sites in Southwest Germany were identified as positive. The most common biotopes were old barns, especially with non-concrete ground, and close to humans and animals. Generally, it appears that these sand flies are associated with a relatively stable and calm environment. The occurrence of phlebotomine sand flies in Southwest Germany is expected to be larger than the number of collected specimens suggests. This reinforces the need for further surveillance in suitable regions in Germany. Furthermore, there is an urgent need to clarify the vector competence and capacity of *P. mascittii.* Global warming might lead to an extension of the distributional area of sand flies and therefore an increase in the risk for sand fly-borne infections in Southwest Germany.

## Supplementary information

**Additional file 1: Table S1.** Collection sites, date of collection and number of captured *Ph. mascittii* (male (m) and female (f)) of the entomological field study in Southwest Germany during 2015–2018 (*collection sites with data logger in 2018).

## Data Availability

Data supporting the conclusions of this article are included within the article and its additional file.

## Abbreviations

BW: Baden-Wuerttemberg
CDC: Center for Disease Control and Prevention
*cox*1: cytochrome *c* oxidase subunit 1 gene
DNA: deoxyribonucleic acid
GFS e.V.: Gesellschaft zur Förderung der Stechmückenbekämpfung e.V
GPS: global positioning system
NRW: North Rhine Westphalia
PCR: polymerase chain reaction
qPCR: quantitative polymerase chain reaction
RNA: ribonucleic acid
RLP: Rhineland-Palatinate
TOSV: Toscana virus
VBD: vector-borne disease
