# Supplementary material for: Phlebotomine sand flies in Southwest Germany: an update with records in new locations
Source: Parasit Vectors. 2020 Apr 21;13:173. doi: 10.1186/s13071-020-04058-6 (PMC7171781; doi:10.1186/s13071-020-04058-6)
Supplement: Supplementary file 1 — Additional file 1: Table S1. Collection sites, date of collection and number of captured Ph. mascittii (male (m) and female (f)) of the entomological field study in Southwest Germany during 2015–2018 (*collection sites with data logger in 2018). [file 13071_2020_4058_MOESM1_ESM.docx]

**Additional file 1: Table S1.** Collection sites, date of collection and number of captured *Ph. mascittii* (male (m) and female (f)) of the entomologic field study in Southwest Germany from 2015-2018; *collection sites with data logger in 2018

| **No.** | **Collection sites** | **Date of collection** | **No. of traps** | **Coordinates** | ***Ph. mascittii***  **(female/male)** | **Site description** | **Potential hosts** |
| --- | --- | --- | --- | --- | --- | --- | --- |
| **1** | Neuenburg | 12.08.2015 | 2 | 47.8088333°N 7.5625°E | 1m | Intra-urban barn of a farm with clay soil near human dwelling, cats, cattle | Dogs, cats, rodents |
| **2** | Obereggenen1 | 12.08.2015  13.08.2015  14.08.2015  15.08.2015  16.08.2015  17.08.2015  19.08.2015  20.08.2015 | 3  3  3  3  3  3  3  3 | 47.75671°N 7.64579°E | 1f  1m  2f  1f 2m  1f  1f  0  0 | Unused barn of an old farm divided into two parts, former cow barn with clay soil ground and mud walls, near human dwelling, cats, hedgehog, field-mice, bats | Cats, rodents, lizards |
| **3** | Obereggenen2 | 12.08.2015  13.08.2015  14.08.2015  15.08.2015  16.08.2015  17.08.2015  19.08.2015  20.08.2015 | 2  2  2  2  2  2  2  2 | 47.75690°N 7.64497°E | 2f  0  0  0  0  0  1m  0 | barn of a former farm with clay soil near human dwelling, cats; Storage and mostly empty | Cats, rodents, lizards |
| **4** | Vögisheim1 | 13.08.2015  14.08.2015  15.08.2015  16.08.2015  17.08.2015  19.08.2015  20.08.2015 | 2  3  2  2  2  2  2 | 47.79152°N 7.62189°E | 0  0  1m  0  0  0  0 | barn of a former farm with clay soil near human dwelling, cats and unused and storage of machines | Cats, rodents, lizards |
| **5** | Vögisheim2 | 13.08.2015  14.08.2015  15.08.2015  16.08.2015  17.08.2015  19.08.2015  20.08.2015 | 2  2  2  2  3  3  3 | 47.79190°N 7.62096°E | 0  0  0  0  0  1f 1m  0 | barn of a former farm with clay soil near human dwelling, cats | Cats, rodents, bats, lizards |
| **6** | Hochstadt1 | 19.07.2017 04.08.2017  17.08.2017 31.08.2017 | 5  4  4  4 | 49.23919°N 8.20601°E | 4f 3m  2f  0  0 | Wooden barn with clay soil ground and partially brick and mud walls, near human dwelling and cats, storage of working bank and machines | Cats, rodents, lizards |
| **7** | Hochstadt2 | 17.08.2017 31.08.2017 | 2  2 | 49.23975°N 8.20592°E | 1f 1m  1m | Barn with concrete floor and parts of clay soil ground and brick walls; only storage of equipment, old furniture | Dogs, cats, rodents |
| **8** | Essingen | 03.08.2017  17.08.2017 | 2  1 | 49.23466°N 8.17549°E | 1m  0 | Wooden barn of a winemaker farm with clay soil near human dwelling, dogs and chickens, unused barn for machine storage | Dogs, cats, rodents, chicken |
| **9** | Gehrweiler1 | 01.08.2017 | 2 | 49.57579°N 7.77327°E | 1f | Unused Barn with clay soil near human dwelling | Cats, rodents, chicken |
| **10** | Gehrweiler2 | 01.08.2017 | 2 | 49.57545°N 7.77388°E | 3m | Unused Barn with clay soil near human dwelling, storage of machinery | Cats, rodents |
| **11** | Nantzsdietschweiler | 02.08.2017 | 5 | 49.43933°N 7.43451°E | 1f 1m | Barn with clay soil near human dwellings, mostly unused and used as storage of unused items | Cats, rodents |
| **12** | Bornheim | 18.08.2017 | 1 | 49.19159°N 8.16263°E | 1m | Barn with partially clay soil and concreted soil near human dwelling and close to the house; Storage of machinery, old furniture | rodents, Cats |
| **13** | Altdorf | 25.08.2017 | 2 | 49.28648°N 8.21688°E | 1m | Barn with clay soil near human dwelling; cats | Dogs, cats, rodents |
| **14** | Böbingen | 25.08.2017 | 2 | 49.28364°N 8.23447°E | 1f | Barn with clay soil near human dwelling and horses; Storage of old items and furniture | Dogs, cats, horses, rodents |
| **15** | Freimersheim | 18.08.2017  25.08.2017 | 3  3 | 49.26902°N 8.21955°E | 1f  1f | Unused barn with clay soil ground and brick walls near human dwelling, Storage of old machinery | Cats, rodents |
| **16** | Hartheim | 09.08.2017  15.08.2017 | 2  2 | 47.93787°N 7.62700°E | 1m  0 | Barn with clay soil near human dwelling and cats | Cats, rodents |
| **17** | Bremgarten | 22.07.2017 09.08.2017  15.08.2017 | 4  3 | 47.91814°N 7.62168°E | 1f 1m  6f 1m  2f | Barn with clay soil and parts of clay soil near human dwelling, houses and chickens | Chicken, cats, rodents |
| **18** | Eschbach | 09.08.2017 | 2 | 47.88854°N 7.65922°E | 1f | Old barn with clay soil; unused and empty | Cats, rodents |
| **19** | Obereggenen1 | 20.07.2017  21.07.2017 13.08.2017 15.08.2017 | 4  3  3  3 | 47.75671°N 7.64579°E | 3f 3m  1f 2m  2f  1m | Wooden barn with clay soil near human dwelling | Cats, rodents, lizards |
| **20** | Obereggenen2 | 20.07.2017  21.07.2017 13.08.2017 | 2  2  2 | 47.75690°N 7.64497°E | 0  1f  0 | Unused barn of an old farm divided into two parts, former cow barn with clay soil ground and mud walls, near human dwelling and partial reconstruction to a garage | Cats, rodents, lizards |
| **21** | Obereggenen3 | 15.08.2017 | 2 | 47.75772°N 7.64606°E | 1f | barn of a former farm with clay soil near human dwelling, cats; Storage and mostly empty | rodents,  cats, hedgehog, field-mice, bats |
| **22** | Vögisheim1 | 20.07.2017  13.08.2017 | 1  1 | 47.79152°N 7.62189°E | 1f  0 | barn of a former farm with clay soil near human dwelling, cats and unused and storage of machines | Cats, rodents, bats, lizards |
| **23** | Hochstadt1* | 30.06.2018 19.07.2018 20.07.2018 28.07.2018 17.08.2018 08.09.2018 01.09.2018 25.08.2018 21.09.2018 | 2  2  2  2  2  2  2  2  2 | 49.23919°N 8.20601°E | 0  2f 4f 1f  0  0  0  0  0 | Wooden barn with clay soil ground and partially brick and mud walls, near human dwelling and cats, storage of working bank and machines | lizards |
| **24** | Hochstadt2 | 19.07.18  20.07.18  28.07.2018  17.08.2018  08.09.2018  01.09.2018  25.08.2018  20.09.2018  21.09.2018 | 2  2  2  2  2  2  2  2  2 | 49.23975°N 8.20592°E | 0  1f 1m  0  1m  0  0  0  0  0 | Barn with concrete floor and parts of clay soil ground and brick walls; only storage of equipment, old furniture | Cats, rodents |
| **25** | Essingen* | 15.07.18  24.07.2018  05.08.2018  01.09.2018 | 2  2  1  1 | 49.23466°N 8.17549°E | 0  1m  1m  0 | Wooden barn of a winemaker farm with clay soil near human dwelling, dogs and chickens, unused barn for machine storage | Dogs, cats, rodents |
| **26** | Gehrweiler2* | 16.08.2018 | 2 | 49.57545°N 7.77388°E | 1f | Unused Barn with clay soil near human dwelling, storage of machinery | Dogs, cats, rodents, chickens |
| **27** | Altdorf* | 15.07.2018  24.07.2018  03.08.2018  17.08.2018  08.09.2018  01.09.2018  20.09.2018 | 3  2  3  2  2  2  2 | 49.28648°N 8.21688°E | 0  2f 1m  2f 1m  1f 1m  0  0  0 | Barn with clay soil near human dwelling; cats | Cats, rodents |
| **s28** | Böbingen* | 15.07.2018  24.07.2018  03.08.2018  08.09.2018  01.09.2018  20.09.2018 | 2  2  2  2  2  2 | 49.28364°N 8.23447°E | 0  1f  0  0  0  0 | Barn with clay soil near human dwelling and horses; Storage of old items and furniture | Dogs, cats, horses, rodents |
| **29** | Freimersheim* | 15.07.2018  24.07.2018  28.07.2018  05.08.2018  17.08.2018  08.09.2018  01.09.2018  20.09.2018  21.09.2018 | 3  3  3  3  3  2  2  2  2 | 49.26902°N 8.21955°E | 1f 2m  1f  2m  1f  0  0  0  0  0 | Unused Barn with clay soil ground and brick walls near human dwelling, Storage of old machinery | Horses, dogs, cats, rodents |
| **30** | Kapellen-Drusweiler | 14.07.2018  31.07.2018 | 3  3 | 49.102558°N, 8.028127°E | 1f  0 | Open Barn with clay soil ground and wood/brick walls very close to human dwelling, Storage of old machinery | Cats, rodents |
| **31** | Eschbach | 10.08.2018 | 2 | 47.88854°N 7.65922°E | 1 m | Old barn with clay soil; unused and empty | Cats, rodents, dogs, chicken |
| **32** | Hartheim * | 03.07.2018  28.07.2018  10.08.2018 | 2  1  1 | 47.93787°N 7.62700°E | 0  1 f  0 | Barn with clay soil near human dwelling and cats | Cats, rodents |
| **33** | Bremgarten* | 03.07.2018 28.07.2018 10.08.2018 | 4  3  3 | 47.91814°N 7.62168°E | 7f 6m  5f  2f | Barn with clay soil and parts of clay soil near human dwelling, houses and chickens | Cats, rodents |
| **34** | Obereggenen1* | 03.07.2018  27.07.2018  08.08.2018  12.08.2018  13.09.2018 | 3  3  2  2  2 | 47.75671°N 7.64579°E | 0  6f 2m  2f 1m  1f  0 | Wooden barn with clay soil near human dwelling | Chicken, cats, rodents, lizards |
| **35** | Obereggenen2* | 03.07.2018  27.07.2018  08.08.2018  12.08.2018  13.09.2018 | 2  2  1  1  1 | 47.75690°N 7.64497°E | 1m  3f  2f  2f  0 | Unused barn of an old farm divided into two parts, former cow barn with clay soil ground and mud walls, near human dwelling and partial reconstruction to a garage | Cats, rodents, lizards |
| **36** | Obereggenen3 | 27.07.2018 08.08.2018  09.08.2018  12.08.2018 | 2  2  2  2 | 47.75772°N 7.64606°E | 2f 1m  1f 1m  1m  0 | barn of a former farm with clay soil near human dwelling, cats; Storage and mostly empty | rodents,  cats, hedgehog, field-mice, bats |
| **37** | Isteiner Klotz | 28.07.2018 12.08.2018 | 3  3 | 47.66134°N 7.53010°E | 3f 1m  1f 1m | small cave and niches within a rocky outcrop in a forestry area; national limestone geotope | rodents, lizards |
|  | **total** |  |  |  | **149 (92f 57m)** |  |  |
